# Impact of a learning health system on acute care and medical complications after intracerebral hemorrhage

**DOI:** 10.1002/lrh2.10223

**Published:** 2020-03-10

**Authors:** Koutarou Matsumoto, Yasunobu Nohara, Yoshifumi Wakata, Takanori Yamashita, Yukio Kozuma, Rui Sugeta, Miki Yamakawa, Fumiko Yamauchi, Eri Miyashita, Tatsuya Takezaki, Shigeo Yamashiro, Toru Nishi, Jiro Machida, Hidehisa Soejima, Masahiro Kamouchi, Naoki Nakashima

**Affiliations:** ^1^ Department of Medical Support Saiseikai Kumamoto Hospital Kumamoto Japan; ^2^ Department of Health Care Administration and Management, Graduate School of Medical Sciences Kyushu University Fukuoka Japan; ^3^ Medical Information Center Kyushu University Hospital Fukuoka Japan; ^4^ Medical IT Center Tokushima University Hospital Tokushima Japan; ^5^ Department of Medical Information Saiseikai Kumamoto Hospital Kumamoto Japan; ^6^ Department of Nursing Saiseikai Kumamoto Hospital Kumamoto Japan; ^7^ Department of Neurosurgery Kumamoto University Hospital Kumamoto Japan; ^8^ Division of Neurosurgery Saiseikai Kumamoto Hospital Kumamoto Japan; ^9^ Department of Neurosurgery Sakura Jyuji Hospital Kumamoto Japan; ^10^ Department of Inspection Saiseikai Kumamoto Hospital Kumamoto Japan; ^11^ Center for Cohort Studies, Graduate School of Medical Sciences Kyushu University Fukuoka Japan

**Keywords:** learning health system, oral care, pneumonia, stroke

## Abstract

**Introduction:**

Patients with stroke often experience pneumonia during the acute stage after stroke onset. Oral care may be effective in reducing the risk of stroke‐associated pneumonia (SAP). We aimed to determine the changes in oral care, as well as the incidence of SAP, in patients with intracerebral hemorrhage, following implementation of a learning health system in our hospital.

**Methods:**

We retrospectively analyzed the data of 1716 patients with intracerebral hemorrhage who were hospitalized at a single stroke center in Japan between January 2012 and December 2018. Data were stratified on the basis of three periods of evolving oral care: period A, during which conventional, empirically driven oral care was provided (n = 725); period B, during which standardized oral care was introduced, with SAP prophylaxis based on known risk factors (n = 469); and period C, during which oral care was risk‐appropriate based on learning health system data (n = 522). Logistic regression analysis was performed to evaluate associations between each of the three treatment approaches and the risk of SAP.

**Results:**

Among the included patients, the mean age was 71.3 ± 13.6 years; 52.6% of patients were men. During the course of each period, the frequency of oral care within 24 hours of admission increased (*P* < .001), as did the adherence rate to oral care ≥3 times per day (*P* < .001). After adjustment for confounding factors, a change in the risk of SAP was not observed in period B; however, the risk significantly decreased in period C (odds ratio 0.61; 95% confidence interval 0.43‐0.87) compared with period A. These associations were maintained for SAP diagnosed using strict clinical criteria or after exclusion of 174 patients who underwent neurosurgical treatment.

**Conclusions:**

Risk‐appropriate care informed by the use of learning health system data could improve care and potentially reduce the risk of SAP in patients with intracerebral hemorrhage in the acute stage.

## 1 INTRODUCTION

Stroke is the second leading cause of death and leading cause of disability worldwide.^1^, ^2^, ^3^ Stroke care has undergone substantial changes in recent decades, which have reduced case fatality and disability among patients with stroke.^4^, ^5^, ^6^ Nevertheless, patients with stroke continue to experience various medical complications;^7^, ^8^, ^9^ pneumonia is a common and clinically important complication that may cause early death after stroke.^10^, ^11^, ^12^, ^13^ However, there has been minimal emphasis on the prevention of stroke‐associated pneumonia (SAP) in the acute period after stroke.

There is considerable evidence to support the beneficial effects of oral care in reduction of the risk of pneumonia, especially in older patients;^14^, ^15^ therefore, oral hygiene may be important for the prevention of pneumonia after stroke.^16^, ^17^, ^18^, ^19^ Currently, oral care is considered a promising measure to reduce the risk of pneumonia in patients with stroke. The aim of this study was to determine whether improved oral care could reduce the risk of SAP, following the introduction of risk‐appropriate care derived from the learning health system. For this purpose, we retrospectively investigated data regarding patients with intracerebral hemorrhage who were hospitalized before and after implementation of a learning health system in our hospital.

## 2 METHODS

### 2.1 Study design and setting

We retrospectively analyzed data regarding patients with intracerebral hemorrhage who were hospitalized at Saiseikai Kumamoto Hospital, Kumamoto city, Japan. Saiseikai Kumamoto Hospital is a regional tertiary hospital that serves patients with stroke in southern Japan and provides acute care in a comprehensive stroke care unit. The Institutional Review Board of the hospital approved this study protocol (approval no. 655) and granted a waiver of informed consent for this secondary analysis of anonymous data.

### 2.2 Learning health system in acute stroke care

**FIGURE 1:**
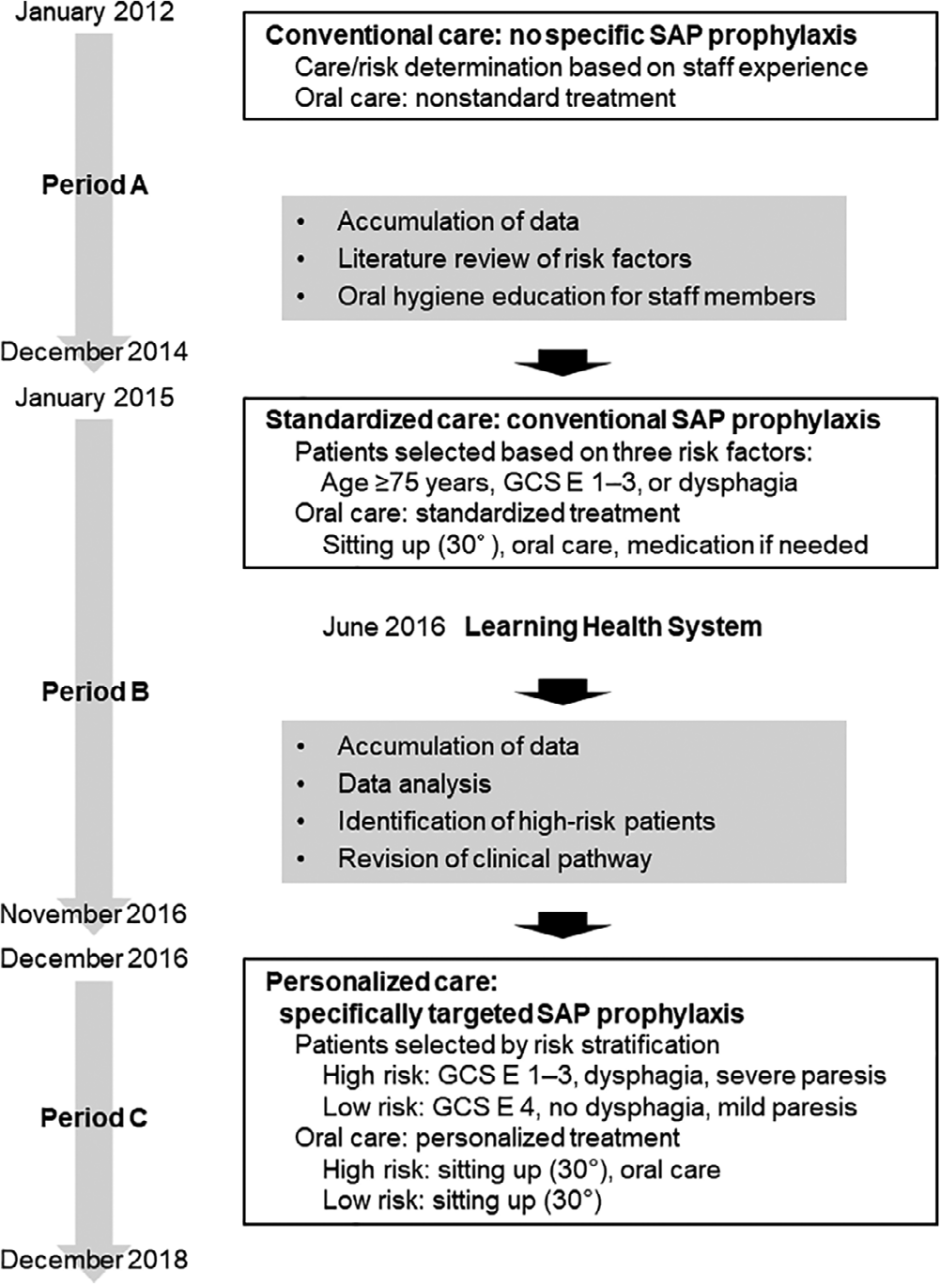
Evolution of oral care. Diagram showing changes in oral care provided and improvement activities in each period. In June 2016, a learning health system was introduced to further improve the quality of oral care. E, eye response component; GCS, Glasgow Coma Scale; SAP, stroke‐associated pneumonia

At our hospital, the stroke care protocol was revised in 2015 to improve oral care and reduce the risk of SAP in daily clinical practice. For further improvement, a learning health system was applied to acute stroke care in 2016. Figure 1 chronicles the evolution of oral care and the actions taken to improve oral care and prevent SAP among patients in our hospital.

#### 2.2.1 Stroke care before implementation of a learning health system

Prior to December 2014 (period A), patients with stroke were provided conventional oral care in accordance with the decisions of nursing staff; these decisions were made on the basis of the clinical experience of the nursing staff and did not involve specific SAP prophylaxis. During this period, oral care was not standardized (Figure 1).

Then, members of the stroke care committee reviewed the literature and arrived at a consensus regarding risk factors for SAP.^11^, ^12^, ^20^, ^21^, ^22^ In January 2015 (period B), the committee provided oral hygiene education to all staff members and instituted standardized care with SAP prophylaxis for patients who were at high risk of SAP, defined as age ≥75 years, Glasgow Coma Scale (GCS) eye response (E) score of 1‐3, or the presence of dysphagia.^11^, ^12^, ^20^, ^21^, ^22^ For convenience in clinical practice, the GCS E score was used as the measure of consciousness level, because all staff members could assess this score without difficulty. GCS E scores were categorized as follows: opening spontaneously (4), to verbal command (3), to pain (2), no eye opening (1).

#### 2.2.2 Learning health system with data collection to identify SAP risk factors

**FIGURE 2:**
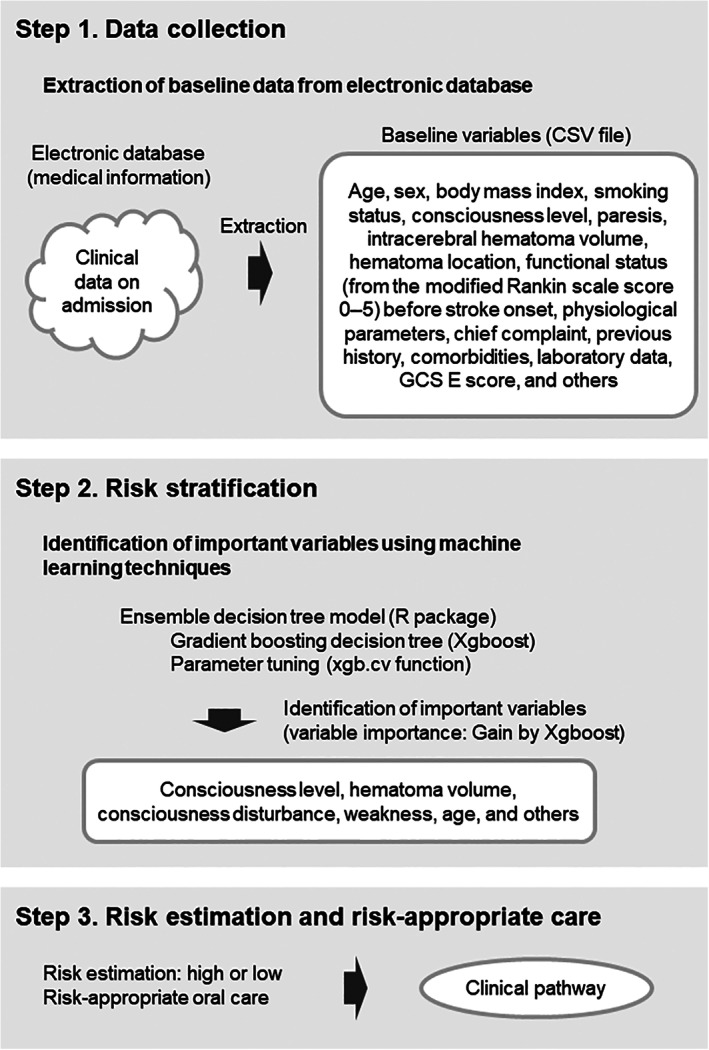
Learning health system. Diagram showing each step of the learning health system used to provide risk‐appropriate oral care for patients with stroke

During the years following introduction of standardized oral care with SAP prophylaxis for high‐risk patients, data were collected and analyzed to identify specific SAP risk factors among patients with stroke in our hospital. In June 2016, a learning health system was implemented to improve routine clinical practice with respect to SAP prophylaxis (Figure 2). To identify risk factors specific to patients with stroke in our hospital, machine learning was used; this constituted a gradient boosting tree, which is a type of ensemble decision tree model.^23^ The electronic database was analyzed to identify baseline characteristics on admission of patients with stroke. In total, 225 clinically important variables were extracted; these included age, sex, body mass index, smoking status, consciousness level on admission, paresis on admission, intracerebral hematoma volume, hematoma location, functional status before stroke onset (using the modified Rankin scale score, 0‐5), physiological parameters on admission, chief complaint, previous history, comorbidities, laboratory data on admission, and GCS E score.

#### 2.2.3 Learning health system with identification of SAP risk factors using machine learning techniques

**FIGURE 3:**
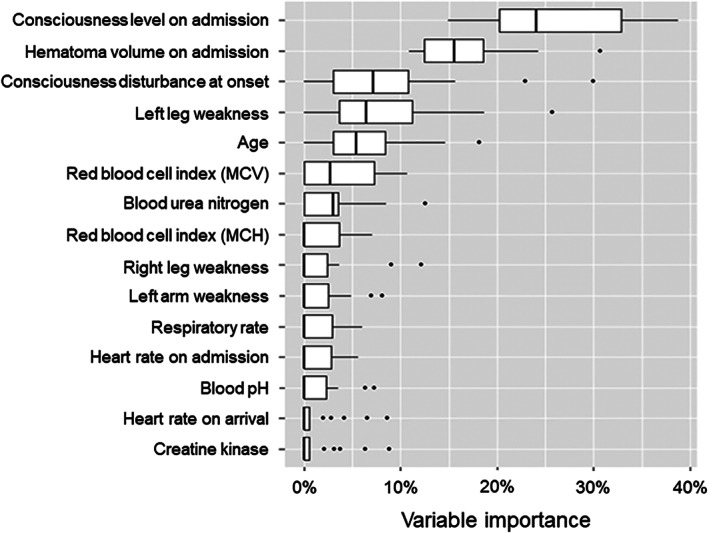
Importance of variables in risk of stroke‐associated pneumonia. Variable importance was estimated by using a gradient boosting decision tree. Box and vertical line in box indicate interquartile range and median, respectively. Horizontal bars indicate 10th and 90th percentiles. MCH, mean corpuscular hemoglobin; MCV, mean corpuscular volume

Through the learning health system, an ensemble decision tree model was used to identify risk factors for the development of SAP during hospitalization. The ensemble decision tree model was used because this model minimizes the influence of nonlinear relationships, interactions, or missing data; moreover, it identifies important variables among a large number of variables. A 10‐fold cross validation was performed to explore the optimal values for parameters in each training set. The R package XGboost (https://cran.r-project.org/web/packages/xgboost/xgboost.pdf) was used to model the gradient boosting decision tree; the xgb.cv function was used for parameter optimization. The importance of each variable was evaluated by the Gain parameter in the gradient boosting decision tree model. Among all variables included in the model, consciousness level on admission was identified as the most important factor; this was followed by hematoma volume, consciousness disturbance at onset, weakness, and age (Figure 3). A few laboratory values, such as red blood cell index and blood urea nitrogen, also were included in the 10 most important SAP risk factors. Based on these findings, the care protocol was revised in November 2016; the new clinical pathway featured risk‐appropriate care.

#### 2.2.4 Learning health system with risk‐appropriate care for specifically targeted SAP prophylaxis

As of December 2016 (period C), the risk of SAP was stratified based on whether patients exhibited consciousness disturbance (GCS E score 4), dysphagia, or severe paresis. Patients were then stratified into high‐ and low‐risk groups based on GCS E score, presence of dysphagia, and degree of paresis (mild vs severe) (Figure 1). In accordance with the clinical pathway, this risk stratification determined the appropriate pattern of oral care (ie, sitting up 30° with or without oral care, combined with specifically targeted SAP prophylaxis for patients at high risk of SAP).

### 2.3 Study patients

**FIGURE 4:**
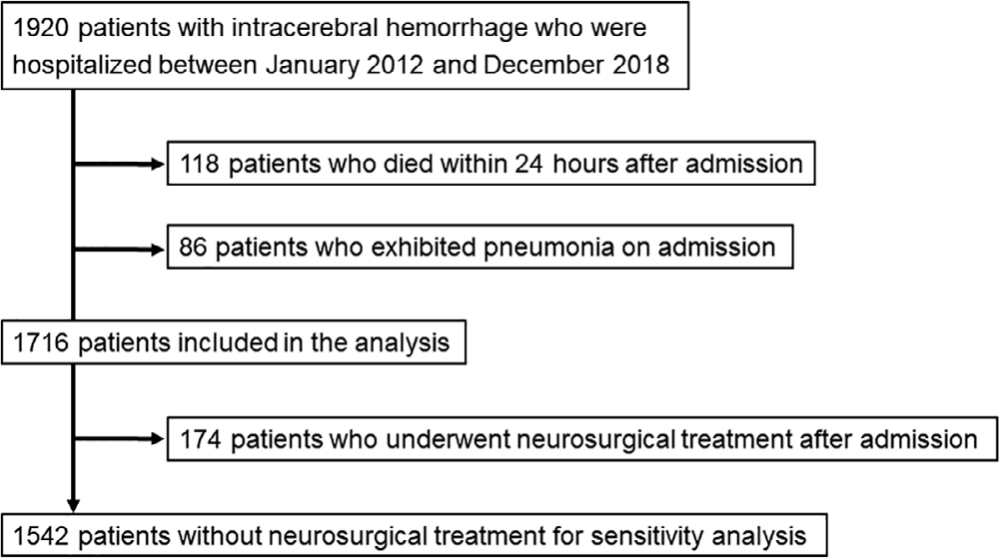
Flowchart of the patient selection process

In this study, we included patients with intracerebral hemorrhage who had been hospitalized before and after implementation of a learning health system at our hospital. Figure 4 depicts the patient selection process. From January 2012 to December 2018, 1920 patients with intracerebral hemorrhage were admitted to our hospital; of these, we excluded 204 patients from this study (118 patients who died within 24 hours after admission and 86 patients with pneumonia on admission [in whom the association between oral care and SAP was unclear]). Finally, we included 1716 patients in the main analysis. For the sensitivity analysis, we also excluded 174 patients who underwent neurosurgical treatment after admission, because they were at risk of ventilator‐associated pneumonia.

### 2.4 Study periods

The study examined three time periods that were defined in accordance with the evolving protocol for oral care, as follows: period A (January 2012‐December 2014), during which conventional empirically driven oral care was provided without a specific SAP prophylaxis protocol; period B (January 2015‐November 2015), during which standardized SAP prophylactic oral care was provided following a literature review; and period C (December 2016‐December 2018), during which data‐driven risk‐appropriate oral care with specifically targeted SAP prophylaxis was provided after introduction of a learning health system.

### 2.5 Quality of oral care

Frequent cleaning of the surfaces of teeth is considered important for oral health.^19^ Therefore, to assess the quality of oral care, we investigated the frequency of oral care in the first 24 hours of admission by quantifying the number of times oral care was provided within 24 hours after admission, and investigated the oral care adherence rate by measuring the proportion of days that oral care was provided ≥3 times per day.

### 2.6 Definition of SAP

In this study analysis, SAP was confirmed based on (a) a clinical diagnosis of pneumonia and (b) a criteria‐based diagnosis.^24^, ^25^ The clinical diagnosis of SAP was made by the treating neurosurgeon, in accordance with the Japanese Respiratory Society guidelines for the management of respiratory infections.^26^, ^27^, ^28^, ^29^ Criteria‐based diagnosis was defined as the presence of SAP on at least two X‐rays, in combination with two or more clinical symptoms that included fever (≥37.5°C), elevated white blood cell count (≥8600/μL), and/or purulent secretions (Miller and Jones' classification P1‐P3); a clinical diagnosis was also required.^26^, ^27^, ^28^, ^29^

### 2.7 Statistical methods

Differences in baseline characteristics among the three periods were analyzed using the *χ* ^2^ test for frequencies, analysis of variance for age and body mass index, and Kruskal‐Wallis test for preadmission modified Rankin scale. Differences in the frequency of oral care within the first 24 hours after admission and in the oral care adherence rate among the periods were assessed by analysis of variance; differences between periods were compared using Tukey's method for multiple comparisons.^30^

First, we estimated the risk of SAP in each patient using a multivariable logistic regression model. The multivariable model included age, sex, consciousness level (GCS E score), ventilator use, and ambulance transport on admission. The receiver operating characteristic area under the curve values in predictive models for clinically diagnosed SAP and data‐based SAP were 0.76 and 0.77, respectively. The Hosmer‐Lemeshow goodness of fit test^31^ demonstrated that the models were well calibrated, because P‐values for the differences between observed and expected event rates were 0.24 and 0.08 for clinical diagnosis and criteria‐based diagnosis of SAP, respectively. Using this multivariable logistic regression model to estimate the risk of SAP, differences in oral care and incidence of SAP were compared among periods.

Logistic regression analysis was also performed to evaluate the associations between each of the three treatment approaches and the risk of SAP. Odds ratio (OR) and 95% confidence interval were estimated after adjustment for age, sex, and consciousness level (as a surrogate of stroke severity). All statistical analyses were performed using R. Two‐way *P*‐values <.05 were regarded as statistically significant.

## 3 RESULTS

### 3.1 Patient characteristics

In total, 1716 patients were included in this study; these patients had a mean age of 71.3 ± 13.6 years, and 903 (52.6%) were men. In total, 1072 patients (62.5%) had a consciousness level of GCS E4 on admission. Table 1 lists the baseline characteristics of the patients for each period, including potential confounders in the association analysis. Age, sex, preadmission functional status, body mass index, and smoking status did not differ among the three periods; however, consciousness disturbance on admission tended to increase in severity among the three periods. In patients who did not undergo neurosurgical treatment, there were no significant differences in baseline characteristics among the three study periods.

**TABLE 1:** Baseline characteristics of the sample population

	Period A	Period B	Period C	*P*‐value
All patients	n = 725	n = 469	n = 522	
Age, y, mean ± SD	71.4 ± 13.4	70.7 ± 13.6	71.7 ± 13.8	.48
Men, n (%)	389 (53.7)	250 (53.3)	264 (50.6)	.53
Preadmission mRS score, median (IQR)	0 (0‐2)	0 (1‐3)	0 (0‐1)	.30
Body mass index, kg/m^2^, mean ± SD	22.4 ± 3.8	22.7 ± 4.5	22.7 ± 4.1	.46
Current smoker, n (%)	202 (27.9)	153 (32.6)	168 (32.2)	.14
Glasgow Coma Scale score, n (%)				
E 4	483 (66.6)	291 (62.1)	298 (57.1)	.007
E 2–3	134 (18.5)	107 (22.8)	118 (22.6)	
E 1	108 (14.9)	71 (15.1)	106 (20.3)	
Patients without neurosurgical treatment	n = 676	n = 424	n = 442	
Age, y, mean ± SD	72.0 ± 13.1	71.3 ± 13.4	72.5 ± 13.7	.45
Men, n (%)	369 (53.1)	223 (52.6)	221 (50.0)	.58
Preadmission mRS score, median (IQR)	0 (0–2)	0 (1–3)	0 (0–1)	.71
Body mass index, kg/m^2^, mean ± SD	22.4 ± 3.8	22.8 ± 4.6	22.5 ± 4.1	.34
Current smoker, n (%)	184 (28.8)	136 (33.9)	131 (31.5)	.14
Glasgow Coma Scale score, n (%)				
E 4	469 (69.4)	279 (65.8)	278 (62.9)	.22
E 2–3	115 (17.0)	86 (20.3)	92 (20.8)	
E 1	92 (13.6)	59 (13.9)	72 (16.3)	

Abbreviations: E, eye response component; IQR, interquartile range; mRS, modified Rankin scale.

### 3.2 Quality of oral care

**FIGURE 5:**
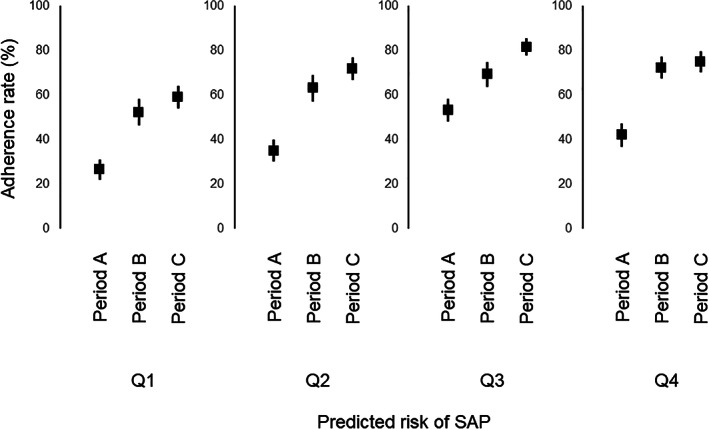
Quality of oral care. Diagram showing oral care adherence rate with respect to predicted risk of stroke‐associated pneumonia (SAP) in periods A‐C. Predicted risk of SAP was estimated from baseline data and stratified into quartiles (Q1‐Q4). Adherence rate was calculated as the proportion of days that oral care was provided ≥3 times per day. Square and error bars indicate mean and 95% confidence interval, respectively

Figure 5 shows the oral care adherence rate (provision of oral care ≥3 times per day) in patients stratified into quartiles based on the predicted risk of SAP, among the three periods. The oral care adherence rate appeared to increase during the course of each period. Trend analysis demonstrated an increased adherence rate in each successive period (Table 2). Similarly, the frequency of oral care within 24 hours of onset significantly increased over time, suggesting that oral care improved during the course of each period. Similar trends were observed following exclusion of patients who underwent neurosurgical treatment.

**TABLE 2:** Oral care in each period

	Period A	Period B	Period C	*P*‐value
All patients	n = 725	n = 469	n = 522	
Adherence rate, %	38.9 ± 34.6	63.7 ± 31.8^*^	72.3 ± 27.6^*^	<.001
Frequency within 24 hours	1.1 ± 1.9	2.7 ± 2.1^*^	3.0 ± 2.1^*^	<.001
Patients without neurosurgical treatment	n = 676	n = 424	n = 442	
Adherence rate, %	38.2 ± 34.7	62.4 ± 32.5^*^	70.8 ± 28.3^*^	<.001
Frequency within 24 hours	1.1 ± 1.9	2.7 ± 2.2^*^	3.0 ± 2.1^*^	<.001

*Note*: Data are expressed as mean ± SD. Frequency of oral care within 24 hours of admission indicates the number of times oral care was provided within the first 24 hours after admission. Adherence rate indicates the proportion of days that oral care was provided ≥3 times per day.

* *P* < .05 vs period A by multiple comparisons.

### 3.3 Incidence of SAP

**FIGURE 6:**
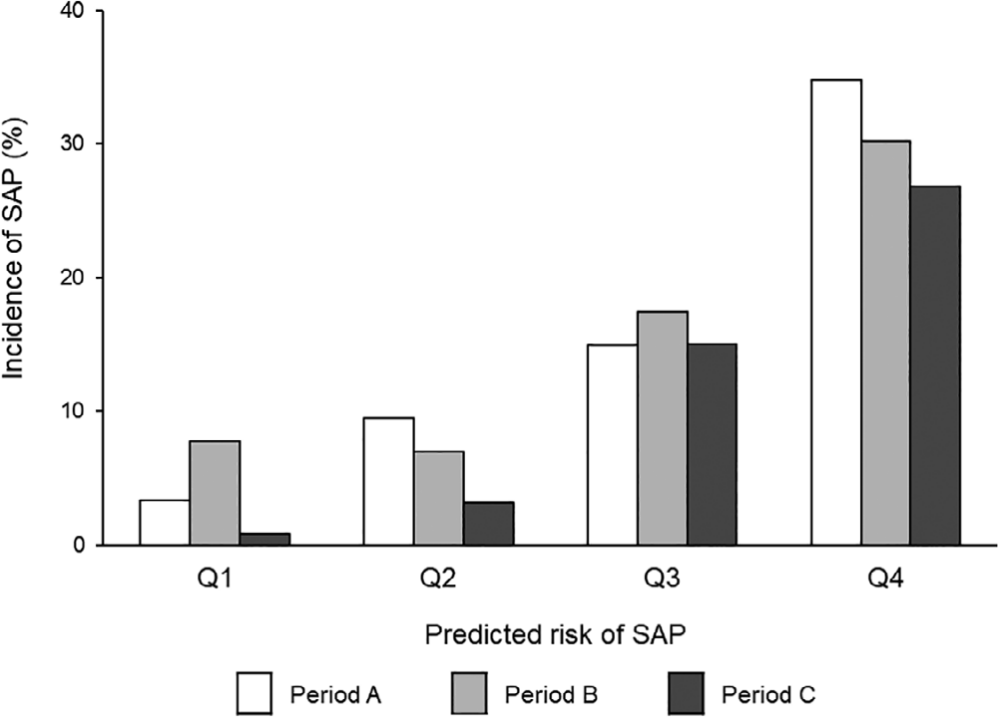
Incidence of stroke‐associated pneumonia (SAP). Diagram showing incidence of SAP with respect to predicted risk of SAP in periods A‐C. Predicted risk of SAP was estimated from baseline data and stratified into quartiles (Q1‐Q4)

Figure 6 shows trends in the incidence of SAP in periods A‐C, with respect to the predicted risk of SAP. The incidence of SAP appeared to decrease in each successive period (Figure 6). After adjustment for confounding factors, the risk of SAP in period B did not differ from that in period A (Table 3); however, the multivariable‐adjusted OR of SAP significantly decreased in period C, with respect to the OR in period A. The risk reduction remained statistically significant when SAP diagnosis was performed using data‐based criteria. In the analysis of patients who did not undergo neurosurgical treatment, the reduced risk of SAP in period C remained, after adjustment for confounders.

**TABLE 3:** Risk of SAP in each period

	Period A	Period B	Period C	*P*‐value
All patients	n = 725	n = 469	n = 522	
Clinical diagnosis				
Event, n (%)	110 (15.2)	72 (15.4)	64 (12.3)	.17
Multivariable‐adjusted OR (95% CI)	1.00 (reference)	1.03 (0.73‐1.44)	0.61 (0.43–0.87)	.01
Criteria‐based diagnosis				
Event, n (%)	102 (14.1)	65 (13.9)	63 (12.1)	.32
Multivariable‐adjusted OR (95% CI)	1.00 (reference)	0.99 (0.69‐1.40)	0.67 (0.46‐0.96)	.04
Patients without neurosurgical treatment	n = 676	n = 424	n = 442	
Clinical diagnosis				
Event, n (%)	94 (13.9)	58 (13.7)	42 (9.5)	.04
Multivariable‐adjusted OR (95% CI)	1.00 (reference)	0.99 (0.68‐1.44)	0.52 (0.34‐0.78)	.004
Criteria‐based diagnosis				
Event, n (%)	87 (12.9)	51 (12.0)	41 (9.3)	.08
Multivariable‐adjusted OR (95% CI)	1.00 (reference)	0.93 (0.63‐1.37)	0.57 (0.37‐0.86)	.01

*Note*: Multivariable model included age, sex, and consciousness level.

Abbreviations: CI, confidence interval; OR, odds ratio; SAP, stroke‐associated pneumonia.

## 4 DISCUSSION

The major findings of this study were as follows: after standardization, oral care improved in terms of early initiation and sustained adherence to an oral care regimen. After implementation of the learning health system, oral care further improved with care planning that was risk‐appropriate, based on data analysis. After standardization, the observed risk of SAP did not differ from the risk that was present in the context of empirically driven oral care; however, the risk of SAP significantly decreased after introduction of the learning health system and revision of clinical pathways to reflect risk‐appropriate oral care planning. In sensitivity analyses, these findings remained statistically significant, despite the use of strict criteria for confirmation of SAP; they also remained statistically significant after exclusion of patients who underwent neurosurgical treatment. Therefore, our use of a learning health system could improve care and lead to favorable outcomes in patients with intracerebral hemorrhage.

### 4.1 Learning health system and quality of care

In our hospital, the efforts to improve care were based on quantitative analysis of real‐world data. In this study, we assessed oral care using two indicators: (a) the frequency of oral care within 24 hours after admission, and (b) the rate of adherence to a pattern of oral care ≥3 times per day during hospitalization. We found both indicators improved after provision of oral hygiene education to staff members and implementation of an oral care protocol for patients with known SAP risk factors. Oral care further improved following our implementation of a learning health system that enabled better identification of risk factors, which resulted in appropriate oral care in routine clinical practice. These results suggest that data‐driven improvement of stroke care is an effective strategy for improvement of the quality of care.

### 4.2 Learning health system and medical complications

This study did not show a difference in the risk of SAP after the standardization of conventional empirically based oral care; however, the risk of SAP was significantly reduced after the implementation of appropriate oral care in the context of the learning health system. This association suggests that risk‐appropriate care can reduce the risk of SAP in patients with acute stroke. Additionally, implementation of the clinical pathway may have played a key role by determining appropriate care based on risk estimation; the frequency of SAP decreased in patients at all risk levels, suggesting that the clinical pathway was able to effectively determine appropriate treatment.^32^, ^33^, ^34^, ^35^

This study did not elucidate a causal relationship between oral care and the risk of SAP. Other factors, such as sitting up at 30°, may have also been involved in reduction of the risk of SAP; however, our findings suggest that data collection and analysis improve stroke care, resulting in enhanced acute‐stage outcomes after stroke. Use of a learning health system is thus a promising strategy to improve care and reduce the risk of medical complications after stroke. Further studies are needed to validate the impact of learning health system use on stroke outcomes.

### 4.3 Study limitations

This study had some limitations. First, it included patients with stroke who were hospitalized in a single stroke center, which may have affected the generalizability of the findings. Second, it did not include analysis of factors that could reduce the risk of SAP during hospitalization; thus, we could not attribute the observed reduction in risk of SAP to the provision of improved oral care or other factors. Third, it involved a treatment bias, as the provision of acute care for stroke (eg, treatment of stroke and the use of antibiotics) was not controlled during the study period. Fourth, this study used real‐world data; therefore, the results may have been limited by missing data, as well as by misclassification of variables and outcomes.

## 5 CONCLUSIONS

Implementation of a learning health system can aid in the provision of risk‐appropriate care for patients with acute intracerebral hemorrhage, thereby reducing risks of medical complications.

## CONFLICT OF INTEREST

The authors declare no conflicts of interest.
